# Evaluation of Hypertension in Pediatric Hemolytic Uremic Syndrome (A Cross‐Sectional Study)

**DOI:** 10.1002/hsr2.71543

**Published:** 2025-11-19

**Authors:** Mahdi Banaei Nourmohammadi, Fahimeh Askarian, Arash Abbasi, Behnaz Bazargani, Daryoush Fahimi, Mastaneh Moghtaderi

**Affiliations:** ^1^ Tehran University of Medical Sciences Tehran Iran; ^2^ Pediatric Chronic Kidney Diseases Research Center, Gene, Cell and Tissue Research Institute, Children's Medical Center Tehran University of Medical Sciences Tehran Iran

**Keywords:** acute kidney injury, *E. coli*, hemolytic uremic syndrome, hypertension, pediatric

## Abstract

**Background:**

Hemolytic uremic syndrome (HUS), particularly the Shiga toxin‐producing *Escherichia coli* (STEC)‐associated subtype, is a major cause of acute kidney injury [AKI] in children. Although hypertension (HTN) is a recognized complication of HUS, its prevalence and related risk factors in these children are particularly poorly studied.

**Objectives:**

This study is designed to determine the prevalence of HTN among children diagnosed with HUS and to identify its potential clinical correlates, including laboratory parameters and cardiac involvement.

**Methods:**

A descriptive cross‐sectional study was conducted on 50 pediatric patients diagnosed with HUS and hospitalized in our tertiary nephrology ward between 2021 and 2024. Clinical and laboratory data, including blood pressure, BMI, renal function markers, and echocardiographic findings, were extracted from medical records. HTN was defined using the AAP Pediatric HTN calculator. Statistical analysis was performed using SPSS v26 software, t‐tests, chi‐square, and Fisher's exact test.

**Results:**

HTN was observed in 48% of our patients. No significant associations were found between HTN and age, sex, BMI, HUS type, urea, creatinine, or hemoglobin levels. However, HTN was significantly associated with hypokalemia (*p* = 0.011) and cardiac involvement (*p* = 0.045). Among patients with AKI, 54.2% had HTN; however, this association was not significant (*p* = 0.402).

**Conclusion:**

Although this study is a small one and was done in a single center, it supports the importance of HTN in patients with HUS. HTN is quite prevalent among children with HUS and shows a significant association with cardiac complications and electrolyte imbalances. These findings indicate the importance of routine blood pressure monitoring and cardiovascular assessment in the management of pediatric HUS. Lifelong follow‐up is recommended for early detection of signs of chronic kidney and cardiovascular sequelae.

## 1 Introduction

Hemolytic uremic syndrome (HUS) is one of the most important childhood disorders and is one of the leading causes of acute kidney injury (AKI) in children. It is clinically defined by the simultaneous occurrence of the triad of microangiopathic hemolytic anemia, thrombocytopenia, and acute renal impairment [1]. Unfortunately, HUS can lead to severe and potentially fatal complications. However, the classic triad defines the syndrome in children; various underlying causes of HUS exist, resulting in differences in clinical presentation, management, and outcomes [2].

HUS primarily affects children under 5 years of age and is recognized worldwide as the most common cause of acute renal failure in infants and young children [3]. In the United States and Western Europe, the annual incidence of STEC‐HUS is approximately 2–3 per 100,000 children under 5 years. In Iran, the prevalence is reported as 79.82 per 1,000,000 children under 15 years [4]. Most children with STEC‐HUS survive the acute phase and recover renal function, although a proportion may develop long‐term renal sequela [5, 6].

Over the past decade, atypical HUS (aHUS) has emerged as a complement‐mediated thrombotic microangiopathy (CM‐TMA), for which novel therapies targeting terminal complement activation are used (e.g., the anti‐C5 monoclonal antibody eculizumab). Nonetheless, there remains a need for prospective, evidence‐based guidelines to determine when to continue or discontinue complement‐inhibiting therapy, especially in patients without identifiable complement gene mutations [7, 8]. While the clinical presentation of STEC‐HUS and non‐STEC‐HUS has been well‐described, hypertension (HTN) remains a critical yet poorly understudied manifestation [4]. Treatment of non‐STEC‐HUS is etiology‐specific.

Several factors, including volume overload and/or kidney injury, can contribute to HTN in HUS patients. Persistent HTN in early childhood is a significant predictor of cardiovascular events, chronic kidney disease, and stroke in adulthood. Accordingly, early detection of HTN and its risk factors in different populations is crucial for the prevention of long‐term complications [9, 10]. Moreover, HTN and pre‐HTN states were identified in 8.4% and 7.8% of children, respectively, underscoring the need for greater attention to this public health issue. In patients with HUS. Cardiovascular dysfunction might be related to some things, such as volume overload, HTN, or electrolyte disturbances, including hyperkalemia. Additionally, primary myocardial involvement has been shown as a potential comorbid feature of HUS (11)

Pediatric HTN is an emerging public health concern and the most important modifiable risk factor for later‐life cardiovascular disease. Numerous studies have reported variable prevalence rates for pediatric HTN [11]. Despite the established link between long‐term HTN and heart failure, evidenced by a meta‐analysis showing that 28.9% of patients with reduced blood pressure developed heart failure, the specific prevalence and impact of HTN in pediatric HUS patients are not well‐defined [12].

Given the low data on the prevalence of HTN in children with HUS in our country and considering the critical impact of elevated blood pressure on clinical outcomes in this population, the aim of this study is specifically to answer the exact prevalence of HTN among children with HUS hospitalized in our tertiary center during 2021–2024.

## 2 Methods and Material

This is a retrospective, analytical cross‐sectional study conducted at a pediatric tertiary center. The study period included all eligible admissions to the nephrology ward of the hospital with a diagnosis of HUS hospitalized during 2021 to 2024. Inclusion criteria were [1]: hospitalization with a confirmed diagnosis of HUS, and [2] availability of complete medical records. Exclusion criteria were [1]: history of known heart disease [2], history of taking medications that affect blood pressure [3], history of liver and biliary diseases. The sampling method was census (non‐probability total enumeration), and all eligible patients during the study period were included, resulting in a total sample size of approximately 50 patients. Of course, the sample size in this study is limited by case availability, and further studies on a larger number of patients are needed to assess and support our findings.

Data were collected retrospectively from hospital records using a structured checklist. The variables extracted included demographic data: age, sex, body mass index (BMI), and clinical data: type of HUS (diarrhea‐associated or non‐diarrhea‐associated), systolic and diastolic blood pressure, and laboratory findings, including renal function tests (serum creatinine, urea, protein), electrolyte levels, and cardiac evaluation: echocardiography findings. All variables were clearly defined before data extraction. Although it is preferable to use ambulatory blood pressure monitoring devices, as this was not possible, blood pressure was measured and recorded with an appropriately sized device according to standard clinical protocols by an expert pediatric nephrologist in our team at the time of admission and 1 week later. The blood pressure was checked three times in the supine or sitting positions, and the highest one was recorded in the patient's data sheet. The diagnosis of HUS was based on established clinical and laboratory criteria. Data entry was performed by trained personnel. To minimize information bias, data were double‐checked for accuracy and completeness. Any discrepancies were resolved by reviewing the original records. Cardiac manifestations of chronic HTN are the development of left ventricular hypertrophy, coronary artery disease, various conduction system diseases, and systolic and diastolic dysfunction of the myocardium, cardiac arrhythmias (especially atrial fibrillation), and congestive heart failure, but in the short term, it may be asymptomatic to heart failure.

### 2.1 Statistical Analysis

Quantitative variables were described using mean and standard deviation, while qualitative variables were summarized as counts and percentages. The normality of distributions was assessed using appropriate statistical tests. Comparisons between groups were performed using independent t‐tests for continuous variables and chi‐square or Fisher's exact tests for categorical variables. A *p*‐value of less than 0.05 was considered statistically significant. All statistical analyzes were conducted using SPSS software, version 26. The study protocol was reviewed and approved by the Ethics Committee of Tehran University of Medical Sciences. Patient confidentiality was maintained throughout the study, and all data were anonymized before analysis.

BMI was calculated using the CDC BMI calculator for children and teens, which covers the age range of 2–19 years [https://www.cdc.gov/bmi/child-teen-calculator/index.html]. For these children, BMI status and percentile were determined according to the calculator's output. For children under 2 years, BMI percentiles were assigned manually using WHO growth charts by sex. These were then entered into SPSS for analysis. According to WHO, for children under 2 years old, < 3rd percentile is defined as Underweight, and Overweight is defined > 97th percentile, and for 2–20 years, based on CDC, is defined as < 5th percentile Underweight, and Obesity is defined ≥ 95th percentile. Also, ≥ 120% of the 95th percentile was defined as Severe obesity (Class II).

HTN was defined based on the initial blood pressure measurement at presentation. Systolic and diastolic blood pressure values were entered into the online AAP Pediatric HTN Guidelines calculator (https://www.mdcalc.com/calc/4052/aap-pediatric-hypertension-guidelines). According to the results, all individuals classified as stage 1 or higher were considered to have HTN, while others were non‐HTN (normal).

AKI was diagnosed using the KDIGO criteria. For patients with at least two serum creatinine values within 48 h, an increase of ≥ 0.3 mg/dL was considered indicative of AKI. If only one value was available after 48 h, AKI was defined as a ≥ 1.5‐fold increase from the baseline. In cases where no prior creatinine value was available, the baseline was estimated using the reverse Schwartz formula: Baseline Cr = [0.413 × height in cm]/120. These two diagnostic strategies were applied based on the availability of data.

Given the variability in normal laboratory ranges across different age groups, age‐specific reference ranges were used to define normal, subnormal, and supranormal values for each test. For some parameters with a single reference range (e.g., hemoglobin [Hb], BUN), age grouping was not necessary.

## 3 Results

All patients diagnosed with HUS are admitted to the nephrology ward, so they have been monitored for body weight and urine output daily, and blood pressure every 6 h. They were taken care of with fluid and salt restriction. Patients with HTN received furosemide 1–2 mg/kg/day with/without amlodipine 0.2–0.6 mg/kg. Day. The baseline characteristics of the patients and results of the blood tests are summarized in Tables 1 and 2. There were no missing data for the main variables of interest. The mean age of children with abnormal blood pressure was 4.95 years (SD = 3.61), while those with normal blood pressure had a mean age of 6.69 years (SD = 3.42). Among children with abnormal blood pressure, 50% were male and 46.2% were female. The mean BMI for children with abnormal blood pressure was 15.87 (Median = 4.15), and for those with normal blood pressure, it was 15.45 (median = 5.14). Typical (diarrhea‐associated) HUS was observed in 76% of cases, and atypical HUS in 24%. The prevalence of hypertension among children with HUS was 48% (24 out of 50), while 52% (26 out of 50) had normal blood pressure (Table 1). Two patients had retinal changes of HTN. One patient had malignant HTN and a seizure, which on the brain CT scan showed PRES (posterior reversible encephalopathy syndrome. During evaluation 12 patients had nephrotic range proteinuria, so 8 patients were discharged with non‐nephrotic range proteinuria that were controlled with ACE inhibitors.

**Table 1:** Baseline characteristics of the all 50 patients.

Variable	Category	*N*	%
Age group (years)	0–1.99	10	20
	2–4.99	11	22
	6–8.99	17	34
	9–13.99	12	24
Age (mean ± SD)			5.3 ± 0.85
Age (range)			0.4–13.58
Sex	Female	26	52
	Male	24	48
BMI category	Underweight	20	40
	Normal	20	40
	Overweight	4	8
	Obese	4	8
	Severely obese	2	4
BMI (median)			15.4 ± 6.79
BMI (range)			8.26–34.47
Hypertension stage	Normal	26	52
	Stage 1	5	10
	Stage 2	19	38
Type of HUS	Atypical	12	24
	Typical	38	76
	Total	50	100
HUS mortality	Yes	2	4
Cardiac involvement	Yes	7	14
AKI	Yes	24	48

Abbreviations: AKI, acute kidney injury; BMI, body mass index; BP, blood pressure; HUS, hemolytic uremic syndrome.

There was no statistically significant relationship between blood pressure and age (*p* = 0.087), sex (*p* = 0.786), BMI (*p* = 0.759), or type of HUS (*p* = 0.243). Abnormal levels of sodium, calcium, and phosphorus were observed in several patients, but only potassium was significantly associated with HTN. Most patients (87.8%) had elevated urea, and 90% had elevated creatinine. Many of patients with AKI (73%) needed dialysis which 80% peritoneal dialysis and 20% hemodialysis. Peritoneal dialysis was done continuously, and hemodialysis three or two times in week until urine flow was established. Low hemoglobin levels were present in 80% of children, with 47.5% of those also exhibiting abnormal blood pressure. Two children (4%) died during the study period; both of them had anuria and HTN (Table 2). 15 patients of 24 patients with hypertension (65%) were discharged with medication for HTN.

**Table 2:** Blood test results of the all 50 patients.

Test	Category	N	%	Reference range
Hemoglobin	Low	40	80	11–16 g/dL
	Normal	10	20	
BUN	High	43	87.8	5–18 mg/dL
	Normal	6	12.2	
Creatinine	Low	1	2	Age‐adjusted
	Normal	4	8	
	High	45	90	
Sodium	Hyponatremia	26	52	134–143 mmol/L
	Normal	21	42	
	Hypernatremia	3	6	
Calcium	Hypocalcemia	33	68.8	8.8–10.8 mg/dL
	Normal	15	31.2	
Potassium	Hypokalemia	7	14	Age‐adjusted
	Normal	39	78	
	Hyperkalemia	4	8	
Phosphorus	Normal	28	58.3	Age‐adjusted
	Hyperphosphatemia	20	41.7	
Albumin	Low	23	57.5	3.5–5.2 g/dL
	Normal	17	42.5	

Regarding the association between blood pressure and laboratory findings such as urea, creatinine, or hemoglobin; we did not find any significant relation except for potassium levels (*p* = 0.011). Also, 14% of our patients had heart failure and 85.7% of these had abnormal blood pressure. So, the association between heart failure and HTN was statistically significant (*p* = 0.045). None of these patients had left ventricular hypertrophy on echocardiography. Among patients with AKI, 54.2% had abnormal blood pressure with no statistically significant relation (*p* = 0.402).

## 4 Discussion

This cross‐sectional retrospective study investigated the prevalence of hypertension among children diagnosed with HUS admitted to the nephrology ward of the Children's Medical Center during 2021–2024. Among the 50 children included, the prevalence of hypertension was 48%. The study found no statistically significant associations between hypertension and age, sex, BMI, type of HUS, serum urea, creatinine, hemoglobin, or most electrolyte disturbances, except for potassium levels and the presence of cardiac involvement, including: heart failure, mild pericardial effusion, and ectasia of the coronary artery and aorta, which were significantly associated with abnormal blood pressure.

The observed prevalence of HTN (48%) in our study is consistent with previous reports but varies across populations and methodologies. For example, a study in Peru reported a prevalence of 52.6% [13], while a Norwegian study found a rate of 37.5% among children with HUS [14]. A systematic review and meta‐analysis in Iran reported a higher prevalence of 79.82% in children under 15 years [9], and Alletto et al. (2023) reported a prevalence of 54.5% [15]. These differences may be attributed to variations in study design, diagnostic criteria, and patient populations. According to this study, 54.2% of children with AKI had HTN (*p* = 0.402). These results align with previous research [16, 17, 18, 19]. In our study, two cases died (4%), which has a similar rate in comparison with previous studies [14, 15, 20, 21].

The majority of our patients had typical [diarrhea‐associated] HUS, which is consistent with previous studies in both Iran and other countries [9, 22, 23, 24]. Among typical cases, 52.6% had HTN. The association between HUS type and HTN was not statistically significant (*p* = 0.243) in line with several published reports. Our study also found a significant association between cardiac involvement, such as heart failure and hypertension, with 85.7% of children with heart failure exhibiting abnormal blood pressure. This is consistent with Sanders et al. (2021) and Freedman et al. (2023), who reported cardiac manifestations in HUS, including pericardial disease and cardiomyopathy [4, 25].

In terms of demographic factors, our findings align with previous studies indicating no significant association between hypertension and sex or age. However, some studies, such as Byrne et al. (2023), have reported associations between age and blood pressure [22], and Akbari et al. (2017) observed a higher prevalence in males, though not statistically significant [11, 26]. Regarding BMI, our findings did not show a significant association, consistent with some reports, although other studies have found higher rates of hypertension among children with increased BMI [20, 26].

Laboratory findings, including elevated urea and creatinine, low hemoglobin, and electrolyte disturbances, were common but generally not significantly associated with hypertension, except for potassium. These findings are in agreement with Freedman et al. (2023) and Cody et al. (2019), who also identified azotemia, proteinuria, and electrolyte abnormalities as key features of HUS [4, 16, 27].

Key limitations include the cross‐sectional design, which prevents causal inference, and reliance on retrospective chart review, which may introduce measurement and data entry errors. Our findings highlight the high prevalence of hypertension among children with HUS and underscore the importance of routine blood pressure monitoring and management in this population, particularly in those with cardiac involvement or electrolyte disturbances. While our results are generally consistent with previous studies, differences in prevalence rates and associated factors emphasize the need for larger, multicenter, and prospective studies to better understand the epidemiology and risk factors for hypertension in pediatric HUS. Future research should include longitudinal follow‐up to assess long‐term outcomes and complications, as well as the evaluation of interventions aimed at reducing the burden of hypertension and its sequelae in children with HUS.

## 5 Conclusion

Children diagnosed with hypertension have a higher associated long‐term risk of major adverse cardiovascular events compared with controls, and improved detection, follow‐up, and control of pediatric hypertension may reduce the risk of adult cardiovascular disease. Our findings demonstrate the need for vigilant monitoring and management of blood pressure and cardiac and renal function in pediatric HUS patients. Future research is suggested to elucidate the underlying mechanisms and should consider to develop effective preventive and therapeutic and long‐term surveillance programs to mitigate chronic complications associated with HUS. The major limitations of our study were a small sample size, a single‐center study, and the lack of ABPM equipment.

## Author Contributions

This study is a project done by Mahdi Banaei Nourmohammadi, supervised by Mastaneh Moghtaderi. Fahimeh Askarian, Daryoush Fahimi, Behnaz Bazargani, Arash Abbasi, and Mastaneh Moghtaderi provided cases to the study. All authors discussed the results and contributed to data analysis and the final manuscript preparation. All authors have read and approved the final version of the article. Dr. Mastaneh Moghtaderi had full access to all of the data in this study and takes complete responsibility for the integrity of the data and the accuracy of the data analysis.

## Ethics Statement

The study protocol was reviewed and approved by the Ethics Committee of Tehran University of Medical Sciences [Ethics Code: IR.TUMS.CHMC.REC.1403.024]. Written informed consent was waived by the Ethics Committee due to the retrospective nature of the study and use of anonymized data. Patient confidentiality was strictly maintained. CONSORT reporting guidelines for observational research were used and adhered to throughout the study.

## Conflicts of Interest

The authors declare no conflicts of interest.

## Transparency Statement

The lead author Mastaneh Moghtaderi affirms that this manuscript is an honest, accurate, and transparent account of the study being reported; that no important aspects of the study have been omitted; and that any discrepancies from the study as planned (and, if relevant, registered) have been explained.

## Data Availability

The data that support the findings of this study are available from the corresponding author upon reasonable request.
